# Hidden in Plain View: Discovery of Chimeric Diabetogenic CD4 T Cell Neo-Epitopes

**DOI:** 10.3389/fimmu.2021.669986

**Published:** 2021-04-27

**Authors:** Brendan K. Reed, John W. Kappler

**Affiliations:** ^1^ Research Division, Barbara Davis Center for Diabetes, University of Colorado, Aurora, CO, United States; ^2^ Department of Immunology and Genomic Medicine, National Jewish Health, Denver, CO, United States; ^3^ Department of Immunology and Microbiology, University of Colorado, Aurora, CO, United States; ^4^ Biochemistry and Molecular Genetics, University of Colorado, Aurora, CO, United States

**Keywords:** antigen presenting cell, transpeptidation, immune tolerance, type 1 diabetes mellitus, chimeric peptide, CD4 T cell, beta cell, antigen

## Abstract

The T cell antigens driving autoimmune Type 1 Diabetes (T1D) have been pursued for more than three decades. When diabetogenic CD4 T cell clones and their relevant MHCII antigen presenting alleles were first identified in rodents and humans, the path to discovering the peptide epitopes within pancreatic beta cell proteins seemed straightforward. However, as experimental results accumulated, definitive data were often absent or controversial. Work within the last decade has helped to clear up some of the controversy by demonstrating that a number of the important MHCII presented epitopes are not encoded in the natural beta cell proteins, but in fact are fusions between peptide fragments derived from the same or different proteins. Recently, the mechanism for generating these MHCII diabetogenic chimeric epitopes has been attributed to a form of reverse proteolysis, called transpeptidation, a process that has been well-documented in the production of MHCI presented epitopes. In this mini-review we summarize these data and their implications for T1D and other autoimmune responses.

## Introduction

In the Non-Obese Diabetic (NOD) mouse model of T1D, a variety of CD4 T cell clones or T cell hybridomas were prepared that responded to antigens within the secretory granules of the beta cells of pancreatic islets of Langerhans (1, 2). In some cases, the protein source of the stimulatory activity was identified (1, 3), but in others, no target could be identified. A particularly stringent test for the relative contribution of these T cells to the disease came from introducing the T cell clones into immunodeficient NOD-SCID mouse lacking T cells and observing whether the clone was sufficient to induce T1D (4). A number of T cell clones failed this test, but others, originally isolated by investigators at the Barbara Davis Center (BDC) (1, 2) were very active. It has now taken an effort of more than two decades to identify the functional peptide epitopes recognized by these BDC CD4 T cells. This work has now identified diabetogenic CD4 T cell epitopes derived in part from three beta cell proteins - insulin, chromogranin A (ChgA) and islet amyloid polypeptide (IAPP). In each case a fusion to the N- or C-terminus fragment of these proteins to a peptide from another or the same protein was required to construct a fully stimulatory chimeric epitope. We begin with a short review of how these three altered antigenic epitopes were discovered.

## Identification of Epitopes for Diabetogenic T Cells

### Insulin

Insulin has become recognized as a major CD4 T cell target in T1D in humans and the NOD mouse model of the disease, reviewed in (5). In the 1990’s at the BDC, a series of CD4 T cells clones were produced from the NOD mouse, including the prototypical BDC-12-4.1 and BDC-12-4.4 clones, that were reactive to a peptide from the insulin B chain, B:9-23, presented by the NOD MHCII allele, IA^g7^ (2, 3). Many of these clones were diabetogenic when introduced into NOD mice. Subsequently, the BDC (6) and other institutions (7, 8) went on to produce many other T cell clones and T cell hybridomas reactive to this peptide. Later, similar T cells were identified in human T1D reactive to the same peptide presented by human DQ8 (9–11). In IA^g7^ (12, 13), HLA-DQA1*03:01/DQB1*03:02 (HLA-DQ8) (14) and other MHCII alleles, the core of the peptide binding groove accepts 9 amino acids in the p1 to p9 positions. Therefore the 15 amino acid B:9-23 peptide could theoretically bind in multiple positions or “registers” (Regs) in the MHCII groove, each with different amino side chains interacting within anchoring pockets in the binding groove versus appearing on the surface for T cell receptor (TCR) recognition. Three registers for this peptide have been studied the most. The 9 amino acid cores of these epitopes are: Reg1-B:12-20, VEALYLVCG, Reg2-B:13−21, EALYLVCGE and Reg3-B:14-22, ALYLVCGER. Several studies proposed Reg1 or Reg2 bound epitopes as the relevant peptide register for two groups of B:9-23 reactive T cells (termed Type B and Type A, respectively after the nomenclature of the Unanue laboratory) (7, 8, 15). However, we performed many experiments that have led us to conclude that the relevant register for both types of T cells is actually Reg3.

To study these registers, we made versions of the B chain peptide in which the amino acids predicted at the p1 and p9 positions in the various registers were mutated to optimize binding to IA^g7^ in that register, but to inhibit T cell recognition if bound in a different register (16). When tested with the BDC-12-4.1 T cell, as well as others reported to respond to the peptide in bound in Reg1 and Reg2 (7), only the peptide forced to bind in Reg3 stimulated these T cells. Additional experiments established that the key modification to the peptide for Reg3 binding was the mutation of B:22R to E at p9, thus changing a very unfavorable amino acid for the IA^g7^ p9 pocket for an optimal acidic one (12, 13, 17). Similar experiments with human T cells responding to B:9-23 bound to HLA-DQ8 established that the B:22R to E mutation at p9 greatly improved T cell reactivity. Eventually, crystal structures of the peptide bound to IA^g7^ or DQ8 confirmed the Reg3 binding of the mutated peptide (10, 11). This modified peptide has been used as a tolerogen for *in vivo* prevention of T1D in NOD mice (18).

We subsequently performed other experiments (19) showing that, while the peptide with p9R to E mutation strongly stimulated Type A T cells, it remained a weak antigen for Type B T cells. This was eventually tracked to interference of the Type B T cell responses by the exposed side chain of B:21E at p8 in Reg3. Combining the p9R to E with a p8E to G mutation to remove the interfering p8 side chain created a strong agonist for Type B T cells, but reduced the Type A T cell responses. Subsequently, crystal structures of these complexes and of Type A and Type B TCRs bound to them explained the Type A vs. Type B discriminating activity of the Reg3 mutations (10, 11). These studies also showed that, for a subset of Type B T cells, changing the p8E to V or L, rather than G, resulted in epitopes that were even stronger stimulators, sometimes even 100-fold better than the p8G modified version (11). Therefore, creating the appropriate CD4 T cell epitopes from the B:9-23 peptide required modifications of the peptide at B:22R (p9) to greatly improve IA^g7^ binding and sometimes also at B:21E to greatly improve TCR interaction.

### Chromogranin A

A similar multi-decade effort led to the identification of the epitope for other T cells identified at the BDC, BDC-2.5 and BDC-10.1 (1, 20). These T cell clones were shown to be extremely diabetogenic in NOD mice (4, 21) and responded to pancreatic islets *in vitro*, but the source of the antigen and the target epitope of these clones eluded researchers for many years. The first clues to its nature came from the identification of stimulatory epitopes for these T cells in various types of peptide libraries (22–25). These independently discovered “mimotopes” eventually pinpointed ChgA as the likely source of the natural antigen (25), since they bore a C-terminal 5 amino acid (p5-p9) motif that was similar to a sequence in ChgA (WSRMD).

A synthetic 9 amino acid ChgA peptide KDRKWSRMD was synthesized, which placed the WSRMD in p5 to p9 positions to mimic the active library mimotope peptides, but this peptide had no activity with the T cells, which we attributed to inhibitory amino acids for T cell recognition (p3R) (25) and IA^g7^ binding (p4K) (17)within the KDRK portion of this peptide. However, we noticed that there was a conserved 14 amino peptide (WE14) (26) released from ChgA during prohormone convertase processing leaving the WSRMD sequence at its C-terminus, while removing the inhibitory amino acids. This peptide stimulated BDC-2.5 and BDC-10.1 weakly, presumably because of the missing p1 to p4 amino acids, but we found that pancreatic islets from mice lacking a functional ChgA gene failed to stimulate these T cell leading us to the conclusion that, while the WE14 peptide was in some way involved in the ChgA derived epitope, a post-translational modification was likely required to make up for the loss at the p1 to p4 positions in the epitope (25).

Delong et al. pursued the idea that the modification was due to the action of the tissue transglutaminase enzyme (TG) on the glutamine within WE14 (27, 28). However, reminiscent of our results with the insulin B:9-23 peptide, we postulated that a more likely modification was one that would change the p1 to p4 positions with optimal TCR and IA^g7^ amino acids. To test this idea, we replaced the natural amino acid extension of WE14 peptide with the N-terminal fragment (RLGL) from our library mimotope peptide (19, 25, 29). This peptide remarkably improved the stimulatory activity of the peptide nearly a million-fold. A crystal structure of this RLGL extended WE14 peptide bound to IA^g7^ confirmed the positions of these amino acids in the peptide binding groove (29). Therefore, we concluded that, in the reciprocal case to that of the insulin B:9-23 derived epitopes, the major epitope for ChgA specific T cells required replacement of the natural ChgA amino acids at the N-terminus, rather than the C-terminus, of the epitope with optimal ones.

### Islet Amyloid Polypeptide (IAPP)

The BDC-6.9 T cell was produced at the BDC at about the same time as the insulin and ChgA specific clones (1). As with the BDC-2.5 and BDC-10.1 clones, it was highly diabetogenic *in vivo* (30), but the source and nature of the epitope was not known. In this case the clue to the source came from the fact that the stimulatory activity was absent in the islets of BALB/c mice (20). Genetic analyses of the stimulatory activity in backcrossed mice mapped it to a section of NOD chromosome 6 and pointed to the IAPP gene as the likely source (30). Several polymorphisms in the IAPP gene coding region between NOD and BALB/c strengthened this idea (31, 32). Disappointingly *in vitro* stimulations at the time with overlapping peptides throughout the IAPP protein failed to identify a stimulating epitope, but experiments in which NOD mice bred to carry the BALB/c genomic region were protected from T1D induction by the BDC-6.9 clone (33) leading to the conclusion that the functional epitope was probably a post-translational modified form of an IAPP peptide. It has taken several decades to confirm this idea.

## Chimeric Peptides Likely Account for the Insulin, ChgA and IAPP Epitopes

The results of the studies above, led to the idea that the functional epitopes for these diabetogenic CD4 T cells were likely post-translational versions of the natural peptides, derived from these proteins. Post-translational modifications of CD4 T cell epitopes had been well-established in other autoimmune diseases, for example, conversion of arginines to citrullines by peptidylarginine deiminases (PADs) in rheumatoid arthritis (34–37) and of glutamines to glutamic acids by tissue transglutaminase (TG) in celiac disease (38). In fact, the presence of these modified amino acids as well as antibodies to the modification or to the modifying enzyme has become diagnostic markers of the diseases.

In T1D, neither of these two types of post translational modification has been established to be a component of the disease driven by the three CD4 T cell specificities discussed here. While Delong, et al. demonstrated an increase in the stimulatory activity of the WE14 peptide after *in vitro* TG treatment (27, 28), the active products of the treatment have not been identified nor did the simple conversion of the glutamine to glutamic acid in the peptide account for the increased activity. Furthermore, the increase in activity was orders of magnitude less than that seen with the library mimotopes (22, 24, 25).

An alternate hypothesis has arisen from studies of post-translationally modified MHCI bound epitopes generated in the proteasome. During the 2000’s a series of studies documented the creation of chimeric MHCI epitopes by the fusion of peptides from the same or different proteins (39, 40)through a form of reverse proteolysis often referred to as “transpeptidation” (41–43). Subsequently, new methods developed to look for these chimeric peptides among those eluted directly from MHCI molecules revealed that they are much more frequent than previously appreciated (44, 45), raising the question that mass spectrometry methods that simply match MHCI bound peptides to sequences in naturally encoded proteins may miss many important MHCI epitopes. These results spurred us (11, 29, 46) and others (33, 47, 48) to test whether synthetic versions of chimeric peptides between pieces of beta cell proteins could create MHCII compatible chimeric epitopes for the diabetogenic CD4 T cells discussed here.

In our studies on the B:9-23 peptide, a scan of the sequence of proinsulin C-peptide revealed short sequences that when synthetically added to the C-terminus of fragments the B:9-23 peptide truncated to B:21 or to B:22 would be predicted to create chimeric peptides with the amino acids at p8 and/or p9 required for stimulation of Type A or Type B insulin reactive T cells (11). In vitro testing of synthetic versions of these chimeric epitopes showed strong activation of the appropriate Type A and Type B CD4 NOD T cells and Type A human CD4 T cells. These results are summarized in **Table 1**. For ChgA, based on the highly stimulatory activity of the RLGL when added to the N-terminus of WE14 (19, 29), we looked in well expressed beta cell granule proteins for similar sequences that could be added to WE14 to make similar complete epitopes predicted to stimulate the BDC-10.1 and/or BCD-2.5 T cell (29). When synthesized, many of these chimeric peptides stimulated BCD-10.1 and or BDC-2.5 T cells, bearing out the predictions (46). These results are summarized in **Table 1**. One of the predicted epitopes involving a fragment of C-peptide with an C-terminal TLAL added to the N-terminus of WE14 has been shown by Delong and colleagues not only to be active, but also present in pancreatic beta cell tumors and in the islets of Langerhans in mouse pancreata (47). This approach of testing candidate fused peptides also turned up the long-sought IAPP-derived epitope for the BDC-6.9 diabetogenic T cell (33). In this case, the same C-peptide fragment ending in TLAL that was used to complete the ChgA WE14 epitope was fused to N-terminus of a peptide released from proIAPP during its natural processing to mature IAPP. This epitope was a very strong agonist for the BDC-6.9 T cell. Importantly the G (p8) from the donor fragment is an R in the corresponding peptide in the BALB/c proIAPP, accounting for the difference between the strains in creating the epitope. As with ChgA, this chimeric peptide has been identified in NOD beta cell tumors and in pancreatic islets (33).

**Table 1:** Chimeric Peptides Derived from Insulin B:9-23, ChgA-WE14 or pro-IAPP.

Acceptor		Donor		Fusion Epitope Sequence	Active T Cell Clone	Synthetic chimeric peptide active *in vitro*	Chimeric peptide found in beta cells	Fused by cathepsin L *in vitro*	Ref
Sequence	Source	Sequence	Source						
VEALYLVCGE	m/h Insulin B:9-23	EVE	mC-peptide	VEALYLVCGEEVE	12-4.1 PCR1-10 I.29 AS150	+	–	–	(11)
"	"	DLQ		VEALYLVCGEDLQ		+	–	–	(11)
"	"	EAE	hC-peptide	VEALYLVCGEEAE	T1D3 T1D4 T1D10	+	–	–	(11)
"	"	EDG		VEALYLVCGEEDQ		+	–	–	(11)
"	"	ELG		VEALYLVCGEELG		+	–	–	(11)
VEALYLVCG	"	GDLQ	mC-peptide	VEALYLVCGGDLQ	8F10 8-1.1 AS91 12-4.4	+	–	–	(11)
"	"	VEQL		VEALYLVCGVEQL	12-4.4 AS91	+	–	–	(11)
"	"	LEVA		VEALYLVCGLEVA		+	–	–	(11)
TLAL	mC-peptide	WSRMDQL	mChgA-WE14	TLALWSRMDQL	BDC-10.1 BDC-2.5 G7W-120	+	+	+	(46, 47)
QLAL	mSecretogranin2	"		QLALWSRMDQL		+	–	–	(46)
RIPV	"	"		RIPVWSRMDQL	BDC-2.5	+	–	–	(46)
TIAL	mSecretogranin3	"		TIALWSRMDQL	BDC-10.1 BDC-2.5 G7W-120	+	–	+	(46)
TLTL	"	"		TLTLWSRMDQL		+	–	+	(46)
ERIL	mChgA	"		ERILWSRMDQL	BDC-2.5	+	–	+	(46)
ILSI	"	"		ILSIWSRMDQL	BDC-10.1 BDC-2.5 G7W-120	+	–	–	(46)
DLAL	"	"		DLALWSRMDQL		+	–	+	(46)
TLAL	mC-peptide	NAARD	NOD lAPP	TLALNAARD	BDC-6.9	+	+	+	(46)
"	"	NAAGD	BALB/c lAPP	TLALNAAGD		+	–	+	(33, 46)

This is a list of chimeric peptides derived from Insulin B:9-23, ChgA-WE14 or pro-IAPP and whether these peptides are capable of stimulating a panel of Diabetogenic T cell clones in vitro, have been discovered in Beta cells, and whether they are capable of being generated by Cathepsin L in vitro. The full length, stimulatory fusion epitope and cognate T cell(s) are listed.

Recently, numerous chimeric epitopes have been reported by others for mouse and human CD4 and CD8 T cells in T1D [reviewed in (49, 50)]. The presence of CD4 and CD8 T cells responding to fusion peptides in mouse and human have now all been described and these findings have bridged the gap in our understanding of the T cell mediated pathogenesis in both the mouse and human diseases (51–53). Additionally, the use of these hybrid peptides as therapeutics to tolerize the cognate T cells and prevent the onset of disease has gained a lot of traction (54), but significant limitations still exist in translating these findings to humans.

## Transpeptidation: The Process of Reverse Proteolysis and Its Implications for MHC I/II Epitopes

These accumulating results with chimeric peptides make it highly likely that addition of amino acids to the N- or C-terminus of fragments of insulin B:9-23, WE14 or IAPP derived peptides create the functional epitopes for the corresponding CD4 T cells in T1D. This conclusion begs the question of what mechanism can lead to the generation of these chimeric epitopes *in vivo*. As mentioned above, the best clues comes from the expanding work on the role of proteasomal transpeptidation in creating many chimeric peptides for MHCI presentation (39, 40, 44, 45, 55–58).

**Figure 1:**
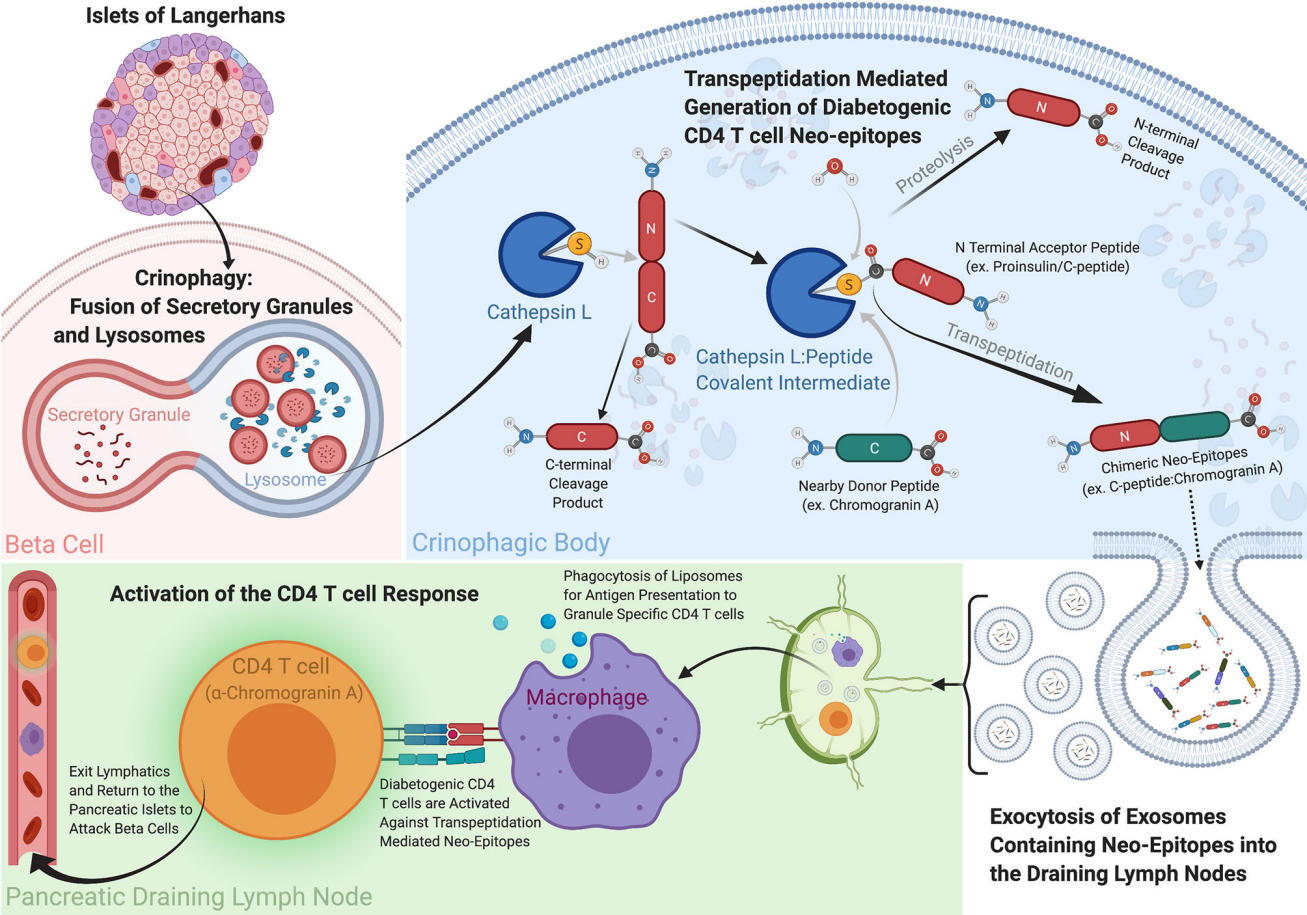
How Transpeptidation in Crinophagic Vesicles Could Create the Chimeric Epitopes Driving T1D. Within the pancreas exist a specialized multicellular network referred to as the Islets of Langerhans. Contained within these islets are the Insulin producing beta cells responsible for maintaining stable blood glucose levels among other neuroendocrine processes. The secretory granules within the beta cells contain prohormones like Proinsulin, Chromogranin A and ProIAPP, and their levels are continually regulated through a catabolic recycling process called crinophagy, whereby secretory granules are fused with lysosomes and their contents are degraded, recycled and secreted. Due to the high concentrations of beta cell hormone donor and acceptor proteins present within these crinophagic bodies, the biochemical conditions are optimal for the reverse proteolysis reaction, transpeptidation to occur. Cathepsin L is a protease capable of cleaving the hormone acceptor peptides (ex. Proinsulin C-peptide) and creating an enzyme linked intermediate complex with the acceptor peptide. Water is generally responsible for breaking this transient bond between the carbonyl carbon of the acceptor peptide and the a sulfur or oxygen in enzyme to complete the digestion, however when high concentrations of a donor peptide with a free N-terminus are present they can outcompete water and generate a new peptide product through transpeptidation. We propose these neo-peptides can be exocytosed and secreted out of the beta cells and to be taken up antigen presenting cells to and presented to diabetogenic CD4 T cells. These peptides are considerably more active than their germline encoded parental counterparts and their presentation can lead to T cell activation and destruction of the beta cells in the islets. This figure was created with Biorender.com.

Transpeptidation is an inevitable side reaction during digestion of proteins with proteases with a catalytic serine, threonine or cysteine in the protease active site [reviewed in (43, 56)] (**Figure 1**). During the protein cleavage reaction these amino acids attack the peptide bond at the cleavage site forming a covalent bond between the oxygen or sulfur in the protease active site and the carbonyl carbon in the peptide bond, while releasing the C-terminal fragment of the digestion. This transient covalent bond is usually broken by water to complete the cleavage by releasing the N-terminal fragment of the digestion and restoring the protease active site, but this bond can also be broken by attack with the N-terminus of a nearby donor peptide restoring a peptide bond and replacing the original C-terminal fragment with a new one to create a chimeric peptide. Transpeptidation is generally a predictable, but minor, side reaction in protease digestions, but its efficiency can be greatly improved by adjusting the conditions present during the proteolysis. Especially effective is a high concentration of the donor peptide in close proximity to the cleavage site and a relatively low concentration of the competing water during the reaction. Under ideal conditions, transpeptidation can be efficient enough to be an important mechanism for natural processing of functional proteins in various organisms (43, 59).

The proteasome has a milieu very favorable for transpeptidation. It contains threonine proteases (60) and has a steady high concentration of cytoplasmic proteins directed into the organelle for degradation [Reviewed in (61)]. It has an encapsulated interior containing low water content. It is the main source of protein digestion products destined to the endoplasmic reticulum for further processing and MHCI loading. These ideal conditions perhaps explain the high proportion of chimeric peptides found in those eluted from surface expressed MHCI molecules (44, 58). While the conditions in the proteasome may be ideal to catalyze these reactions, the spatial constraints of the proteasome have been shown to prefer Cis-splicing events, where internal deletions are made within the same protein, instead of fusion events between two different proteins (Trans-splicing) (62).

## These Diabetogenic Chimeric Epitopes Can Be Produced by Lysosomal Protease Mediated Transpeptidation

A parallel pathway involving lysosomes exists in pancreatic islet beta cells and can be predicted to favor the generation of chimeric peptides. In beta cells, secretory granules have a high concentration of insulin and other proteins, including ChgA and IAPP [Reviewed in (63)]. Convertase proteases in the granules convert the precursor forms of these proteins into their mature, active forms, by releasing protective prohormone fragments, as well as additional active hormone fragments by internal cleavages (64). The number of granules in a beta cell is strictly regulated (63). Therefore, since new granules are constantly being formed, excess granules need to be eliminated to maintain the optimal number. This is accomplished by a form of autophagy called crinophagy (65), in which granules are fused with lysosomes and their proteins denatured and degraded by a variety of enzymes including cathepsins and other cysteine or serine proteases. Thus, ideal conditions for transpeptidation are set up – a high concentration of actively degrading proteins encapsulated in a vesicle with multiple proteases that are capable of the transpeptidation reaction. Exosomes from these crinophagic vesicles carrying antigenic fragments of insulin and other granule proteins can be released from beta cells and into circulation (66), providing a pathway for chimeric peptides to reach the pancreatic draining lymph nodes for activation of diabetogenic CD4 T cells (67) (**Figure 1**). While these findings are considered circumstantial by some, these extracellular vesicles have been shown to carry cargo relevant to T1D in the form of prohormone proteins for both CD4 and CD8 T cells, and miRNAs, all of which have been implicated in multiple facets of the disease [reviewed in (68)]. Since the lysosomal/endosomal pathway in MHCII bearing antigen presenting cells (APCs) is the primary site for proteolytic generation of peptides for MHCII presentation [reviewed (69)], a number of laboratories have studied this antigen processing reaction *in vitro*, by exposing proteins to various lysosomal proteases under lysosomal conditions and testing the products of the digestion for antigenic activity (46, 70–72). We have used this system to see if any of the active chimeric epitopes identified in the beta cells mentioned above could be generated *in vitro* during lysosomal protease digestion of a suitable acceptor protein fragment in the presence of a donor peptide that when fused to a site within the acceptor would form the active diabetogenic epitope (46).

We tested a number of cathepsin proteases, we settled on cathepsin L for these experiments, due to its ability to generate the necessary complimentary Proinsulin acceptor peptide. In looking for an active WE14 containing chimeric epitope, we used a fragment of C-peptide containing the previously documented TLAL sequence discussed above (47), as well as fragments of other granule proteins with embedded sequences that also were active when fused to the N-terminus of WE14 (46). In each case an internal cleavage at the C-terminus of the embedded fragment was required to create a site for transpeptidation fusion to an N-terminal fragment of WE14. Cathepsin L digestions were performed at lysosomal pH with a molar excess of a WE14 donor fragment to favor the transpeptidation reaction.

When the digests were used to stimulate the prototypical NOD WE14-specific CD4 T cells, BDC-10.1 and/or BCD-2.5, five (**Table 1**) were active with one or both T cells (46). Tandem mass spectrometric analysis (MS-MS) of the digests revealed the presence of the predicted chimeric peptide in the digests. Synthetic versions of the identified epitope had the same stimulating specificity as the digests. In each case the identification was further confirmed by showing that the MS-MS fractionation pattern of the synthetic peptide was virtually identical to that seen in the corresponding peptide found in the digest.

The MS-MS analyses also showed that these functional peptides were by no means the only chimeric peptides detected in the digests. Hundreds of additional chimeric peptides were identified involving many combinations of the input acceptor and donor peptides. In the case of joining of the WE14 fragment to sites within the input acceptor peptide precursor, fusions were detected at nearly every position (46), but strikingly, the positions that contained a preferred cathepsin L cleavage sequence were highly favored. A similar digestion with the C-peptide fragment containing TLAL using a donor peptide from NOD proIAPP (NAARD) generated the previously reported functional chimeric peptide for the BDC-6.9 T cell (46). Substituting the equivalent donor peptide from BALB/c IAPP (NAAGD) also generated the predicted chimeric peptide, but as expected this peptide was 10x less active than the NOD derived one.

Cysteine proteases have been implicated in disease resistance in Type 1 Diabetes (73), however further investigation by other groups determined that the effect was indirect, due in part to T cell repertoire changes resulting from Cathepsin L being absent during thymic selection. They observed a 2 fold higher incidence of regulatory T cells in the knockout mice compared to their CatL sufficient counterparts, which they attributed to the disease protection (74). Although splenocytes from NOD mice are capable of mounting a response against the CatlL-/- islets, it is not clear whether the absence of CatL has allowed for a compensatory mechanism whereby alternative proteases are utilized to generate these fusion peptides, or if another protease is responsible for it altogether. To date, we have not discovered another protease capable of generating these fusions other than Cathepsin L.

## Final Thoughts

After a decades-long struggle to understand the structures of the diabetic CD4 T cells epitopes in T1D, the door has cracked open with the discovery of the functional chimeric epitopes and their formation by transpeptidation. As more acceptor-donor pairs are tested with multiple lysosomal proteases, it seems likely that this form of post-translational modification will play an important role in epitope formation in other CD4 T cell driven autoimmune diseases, especially those of other neuro-endocrine tissues containing secretory granules. This phenomenon may also contribute to epitopes for CD4 T cells derived from foreign (viral/bacterial) and tumor antigens. As with MHCI, these MHCII results point out that existing peptides databases for MHCII bound to peptides directly encoded in the genome may be incomplete and need to be updated to include chimeric peptides found directly bound to MHCII molecules. One can hope that the computational methods for identifying chimeric epitopes bound to MHCI molecules can be adapted to those bound to MHCII. The variable lengths of MHCII bound epitopes presents a challenge in approaching this task, but the longer MHCII bound peptides may also be an advantage. They could make it easier to identify independently the N- and C-terminal components of a chimeric peptide among the MS-MS generated b-ion versus y-ion fragments. The similarities between these recent findings within the MHCI and MHCII epitope fields might also provide reason to reexamine old data sets for MHC peptide elutions and reprobe them for their presence of transpeptidation mediated fusions.

## Author Contributions

BR and JK both reviewed relevant literature and drafted the initial manuscript. BR and JK edited and approved the final manuscript.

## Funding

This work was supported by National Institutes of Health grants T32 AI 074491 (BR), P01 AI-118688 (JK). BR was also supported by the Intersect Fellowship Program for Computational Scientists and Immunologists from the American Association of Immunologists.

## Conflict of Interest

The authors declare that the research was conducted in the absence of any commercial or financial relationships that could be construed as a potential conflict of interest.
